# Medium- and Long-Term Immune Responses in the Small Intestine in Piglets from Oral Vaccination against *Escherichia coli*

**DOI:** 10.3390/ani14192779

**Published:** 2024-09-26

**Authors:** Aida Miralles, Guillermo Ramis, Francisco J. Pallarés, Ester Párraga-Ros, Juan Seva

**Affiliations:** 1 Departamento de Anatomía y Anatomía Patológica Comparadas, Facultad de Veterinaria, Universidad de Murcia, 30100 Murcia, Spain; aida.miralles@um.es (A.M.); ester.parraga@um.es (E.P.-R.); jseva@um.es (J.S.); 2 CEFU, S.A., 30840 Alhama de Murcia, Spain; 3 Departamento de Producción Animal, Facultad de Veterinaria, Universidad de Murcia, 30100 Murcia, Spain; 4 Instituto Murciano de Investigación Biosanitaria (IMIB), 30120 Murcia, Spain; 5 Departamento de Anatomía y Anatomía Patológica Comparadas y Toxicología, Universidad de Córdoba, 14071 Córdoba, Spain; fpallares@uco.es

**Keywords:** *Escherichia coli*, oral vaccination, vaccine, intestinal integrity, cytokines, lymphocytes, villus

## Abstract

**Simple Summary:**

Due to the serious problems caused by *Escherichia coli* (*E. coli*) in pigs, it is essential to prevent post-weaning diarrhea and the subsequent growth delay that this entails. In this study, we evaluated intestinal integrity, immune activation and morphometry and cellular composition at the tissue level in different sections of intestine in piglets subjected to oral *E. coli* vaccination in the middle and long term after the vaccine challenge. The results obtained show greater immune stimulation and intestinal integrity in the vaccinated animals compared to the control group with an increase in gene expression in most of the cytokines studied, better parameters in histomorphometry as well as a greater density of intraepithelial lymphocytes.

**Abstract:**

Post-weaning stress, together with *Escherichia coli*, are two of the key factors in the occurrence of post-weaning diarrhea. There are different commercial vaccines that induce immunity at the local or systemic level, improving farm health and avoiding economic losses in the pork industry. That is why the objective of this study was to evaluate the effect of an oral enterotoxigenic *E. coli* F4/F18 vaccine on immunity and intestinal integrity in the middle and long term after inoculation. The gene expression of the biomarkers indicative of cellular infiltration (calprotectin, CAL), tight junction proteins (occludin, OCL; zonulin, ZON; and claudin, CLA) and a panel of proinflammatory (interleukins, IL: IL1α, IL1β, IL6, IL8, IL12p35 and IL12p40; interferons, IFN: IFNα and IFNγ; and tumoral necrosis factor, TNF: TNFα) and anti-inflammatory mediator cytokines (TGFβ and IL10) were analyzed, as well as histomorphology in jejunum and ileum, the cell density of goblet cells, intraepithelial lymphocytes and IgA-producing cells. Differences were observed in ZON, CLA and CAL, with greater gene expression in observed in vaccinated piglets at 42 days post vaccination (dpv) in the ileum. Regarding the expression of cytokines, the vaccinated animals showed significant differences in IL1α, IL6, IL12p35, IL12p40, IFNα, IFNγ, TNFα and TGFβ at 42 dpv in the jejunum or ileum. The villi showed greater height in the vaccinated piglets and the ratio between villus height and crypt depth was significantly greater in the vaccinated group in the jejunum at 84 dpv. The count of IgA-producing cells shows higher values for the unvaccinated group in the ileum, while intraepithelial lymphocytes show a significant increase in both jejunum and ileum in vaccinated piglets. We can conclude that oral vaccination against *E. coli* produces an evident effect, which manifests itself even in the middle and long term after the challenge, including immune response, decrease in antimicrobials usage, better histological structure in intestine and the improvement of performance.

## 1. Introduction

Weaning is one of the most stressful moments in a pig’s life due to the separation of the piglet from the mother and environmental, physical and social changes. The adverse effects that accompany weaning are, among others, a reduction in feed intake and growth and an increase in mortality and morbidity until the piglet’s immune system is fully developed [1]. Intestinal inflammatory processes are increased, as well as structural and functional damage to the intestinal mucosa, so controlling intestinal inflammation at this stage is key to defending intestinal integrity, which in turn is critical for general health and productive performance [2,3,4].

Post-weaning diarrhea due to *Escherichia coli* remains one of the main causes of economic losses for the swine industry [5,6]. The virulence of this pathogen is based on two factors: adhesion fimbriae and exotoxins and endotoxins. Fimbriae allow the adhesion of bacteria to the surface of the intestinal epithelium, the formation of colonies and then, the production of toxins will be what causes the clinical symptoms. There are different types of fimbriae, but in the post-weaning period, the most prevalent are F4 and F18 [6]. Regarding toxin production, enterotoxigenic *E. coli* (ETEC) strains are mainly associated with the production of heat-stable toxin (STa, STb) or heat-labile (LT), which cause secretory diarrhea and play an important role in the early stages of infection [7,8].

Currently, both parenteral and oral *E. coli* vaccines are available. The former are usually based on inactivated bacteria and the latter on live non-pathogenic strains of the bacteria. And among oral vaccines, there are monovalent or bivalent ones, including strains containing adhesion factors F4 or F4 and F18, which have demonstrated a greater local immune response in previous studies [9].

At the intestinal level, active mucosal immunity is required in which the local production of secretory IgA specific for F4 and/or F18 plays an important role to protect pigs against post-weaning diarrhea. The processing of orally administered antigens through gut-associated lymphoid tissue (GALT) produces a local response based on these immunoglobulins which, in the case of *E. coli* colonization, should provide protection against post-weaning diarrhea if antibodies are produced against adhesion elements or exotoxins produced by bacteria [10]. However, there is little information available on the effect of vaccination on intestinal integrity, understood as the integrity of tight junctions and other paracellular junctional elements of the intestinal epithelium, a key element of defense against enteric diseases [9].

The gastrointestinal barrier consists of vascular endothelium, epithelial cell lining, and a mucus layer, as well as a functional immunological barrier from digestive secretions, immune molecules and cellular products such as cytokines, inflammatory mediators and antimicrobial peptides, produced primarily by Paneth cells in the crypts of the small intestine. Considering that intestinal inflammation can negatively affect gastrointestinal function by altering the structure and function of the intestinal mucosa, determining the presence of intestinal inflammatory activity is crucial for the evaluation of the gastrointestinal barrier [11].

The intestinal epithelial layer is composed of epithelial cells connected by intercellular junctions: tight junctions, located in the apical part of the cells, followed by adherens junctions and desmosomes. Tight junctions consist of transmembrane proteins such as occludins, claudins, junctional adhesion molecule (JAM) proteins and intracellular adhesion proteins, such as the zonula occludens proteins [12], and regulatory proteins as zonulin, whose gene expression has been used as a marker of intestinal permeability in pigs [9,12,13,14].

The epithelium of the gastrointestinal tract is lined by a layer of mucin glycoproteins, where bacteria and viruses can become trapped [15]. This mucin layer is a dynamic system that is linked to the immune system through goblet cells that, while producing mucus, simultaneously sample luminal antigens and present them to dendritic cells [16]. On the other hand, intraepithelial lymphocytes can indicate the degree of immunocompetent and immunoregulatory cells that provide an immediate immune response on infected cells [17].

Jointly, mature IgA-producing plasma cells are found in the intestinal lamina propria, which play a crucial role in maintaining the intestinal epithelial barrier and in the development of immune tolerance to commensal intestinal microbes [18]. IgA is the most abundant immunoglobulin in the intestinal mucosa and plays a fundamental role as the first line of defense against toxins and the colonization and invasion of pathogens [17].

At the immune level, activated T lymphocytes have different functions based on the profile of cytokines they secrete at the intestinal level, being crucial for the modulation of the inflammatory response in the gastrointestinal tract [19]. This is why its determination in various individuals could act as biomarkers of intestinal functionality and integrity. Proinflammatory cytokines can induce the endocytosis of tight junction proteins, increasing intestinal permeability [20]. In contrast, anti-inflammatory cytokines play an important role in controlling the duration and magnitude of the inflammatory response [19].

To measure intestinal health, some authors propose the use of biomarkers, substances that can be used as biological indicators. They must be able to be objectively measured and be indicators of a normal biological process, of a pathological state or of a response to pharmacological treatment [21].

Managing piglet intestinal health in the era of antibiotic-reduced production is challenging not only for the pork producer as a result of reduced animal performance and production but also for the veterinarian, due to the implications in animal welfare [22] and in health responsibility regarding the emergence of antimicrobial resistance in humans. In addition to the reduction in the use of antibiotics in animal production, new EU regulations prohibit the use of zinc oxide supplements in feed, all of which increases the need to discover alternative control strategies against digestive diseases in the pig sector [5,23].

It is always interesting to know what response vaccines produce at different times after vaccination. Until now, the effect has been described in the short term, 21 dpv [9], or in the very long term, at the end of the fattening period [24]. But little is known about what happens in the medium and long term, which is where interactions with the pathogen will potentially occur. Following the previous line of research on the local immune response 21 days post-vaccination, the objective of this study was to analyze the effect of the use of an oral enterotoxigenic vaccine of *E. coli* F4/F18 on intestinal health and integrity in the middle (42 days post vaccination) and long term (84 days post vaccination). To this end, the study of the gene expression of tight junction proteins, intestinal immune activation through the expression of pro-inflammatory and anti-inflammatory properties of cytokines, as well as the immunological study of the digestive tract, was proposed. The digestive tract was also studied through the analysis of cellular production of IgA, intraepithelial lymphocytes, goblet cells and intestinal morphometry in the jejunum and ileum of weaned piglets.

## 2. Materials and Methods

### 2.1. Animals and Farm

This study was carried out on a commercial farm in the province of Albacete (Spain). The farm received weaned piglets at 28 ± 3 days of age, and they were placed in flatbed pens containing twenty-three piglets each. The experimental groups were separated in different spaces to avoid the transfer of the live vaccine strain from one to another in case of excretion. Similarly, the flow of workers was designed to provide caregivers first to the control group and then to the vaccinated group, and never in the opposite direction. The study was carried out with 4968 piglets (Landrace–Large White x Duroc) from a farm with no clinical problems related to *E. coli* to avoid interference with the effect of the vaccine. A previous diagnosis was made in 10 piglets that showed the presence of F4 and F18 fimbriae. Rectal swabs with Amies transport medium in plastic vials with hermetic lids were used and sent to the laboratory (Exopol; Zaragoza) for the diagnosis of *E. coli*. Multiplex PCR was performed individually to detect adhesion factor genes, including F4 and F18 fimbriae. Finally, 32 animals (16 not vaccinated and 16 orally vaccinated) were euthanized at 42 and 84 days post vaccination.

Two groups were established: control group (CON) with 2484 piglets and oral vaccine group (V) with 2484 piglets. The animals were kept in the nursery until 84 days post vaccination and were then transferred to the fattening unit; so, all the procedures were carried out in the nursery. The piglets were housed in 2.3 × 2.6 m pens in separate rooms with two corridors and twenty-four stalls. The animals were watered ad libitum by means of a nipple drinker and feed was freely available by means of a feeder with seven openings. Environmental control encompassed two types of heating: underfloor heating in the center of the pen and ambient heating in the whole room by delta tubes. There was cooling for the summer months through foggers in the stables and corridors. Ventilation was forced by fans using upper and lower windows, and the ventilation and temperature control was automated using a weather station and temperature probes.

The composition of the feed consumed by the animals over the whole study period is shown in Table 1.

The animals were fed ad libitum with sufficient feeders for group feeding of all the animals present in the pen. The feeding program included two feeds: a pre-starter for the 3 weeks after weaning and a starter from the 4th week until the end of the nursery period. The compositions of both feeds are shown in Table 1.

All the experimental procedures described in this investigation were carried out under the welfare standards established in the Royal Decree 1135/2002 [25], regarding the minimum standards for the protection of pigs, as well as in the Royal Decree 159/2023, which establishes provisions for the application of the European Union regulations on official controls in the field of animal welfare in Spain. The farms are governed by Royal Decree 306/2020 [26], establishing basic rules for the management of intensive pig farms and amending the basic rules for the management of extensive pig farms.

This trial was approved by the Bioethics Committee of the University of Murcia and authorized by the competent authorities of the Autonomous Community of the Region of Murcia under the code number A13121104.

### 2.2. Vaccination

None of the piglets were treated with antimicrobials within 7 days of vaccination. Twenty-three animals were housed in each pen, with the pens of the two study groups being physically separated. Piglets in the V group were vaccinated with Coliprotec F4/F18 (Elanco GmbH, Monheim am Rhein, Germany), which contains *E. coli*, as live non-pathogenic strains O8:K87 (1.3 × 10^8^ to 9 × 10^8^ UFC) and O141:K94 (2.8 × 10^8^ to 3 × 10^9^ CFU), assuring the presence of adhesins F4ac and F18ac. The piglets were vaccinated at 25 days of life orally and collectively (2 mL per piglet). Vaccination was carried out using plates and when diluting the vaccine, and a dye and stabilizer (Aviblue, Lohmann Animal Health España S.L.U., Huelva, Spain) were used in the water to ensure correct vaccination. This dye makes it possible to monitor whether any animal has been left unvaccinated, in which case the vaccine is administered by drenching.

### 2.3. Growth Performance, Clinical Examination and Consumption of Antibiotics

The 4968 animals were weighed at weaning and at fattening as a group. In addition, a control of the total feed consumed was carried out. With these data, the average daily gain (ADG) and the Feed Conversion Rate (FCR) were calculated. The piglets were monitored daily for their general health and a record of the deaths produced, cause of death and consumption of antibiotics was carried out. Antibiotic consumption was expressed in mg/population correction unit (PCU), which is a system to make antimicrobial use comparable between groups, companies or countries.

### 2.4. Sampling

Animals were euthanized by intravenous thiobarbital overdose and bleeding, the authorized method, which was immediately followed by necropsy, with special attention paid to the assessment of the presence of lesions in the organs, especially to the digestive tract. Finally, a sample of jejunum and ileum were fixed in 4% formalin. An adjacent 100 mg portion was preserved in RNAlater^®^ (Invitrogen, Waltham, MA, USA) for subsequent RNA isolation.

### 2.5. RNA Isolation and cDNA Synthesis

RNA was isolated from 20 mg of tissue samples using the Thermo Scientific Gene JET RNA Purification Kit (ThermoFisher, Waltham, MA, USA) and cDNA was synthesized using the Geneamp RNA PCR Core Kit (Life Technology, Carlsbad, CA, USA) using oligo-dT as primers to obtain cDNA only from mRNA.

### 2.6. Gene Expression for TJ Protein and Cytokines

The gene expression of three tight junction (TJ) proteins was studied: occludin (OCL), zonulin 1 (ZON), and claudin (CLA) and an indicator of the presence of inflammatory cells: calprotectin S100 (CAL); and a panel of nine cytokines as representatives of pro-inflammatory activity (IL1α, IL1β, IL6, IL8, IL12p35, IL12p40, IFNα, IFNγ, TNFα) and two anti-inflammatory cytokines (TGFβ, IL10).

Was performed using the methodology and primers described previously [27] and using β-actin as a housekeeping gene (Table 2). All relative quantifications were performed on jejunum and ileum samples.

### 2.7. Histomorphology and Cell Counts

Formalin-fixed tissues were routinely processed for paraffin embedding. Serial 5 μm thick sections were obtained and stained with hematoxylin–eosin for morphometric measurements and with periodic acid–Schiff for cell counts.

After staining, the histological evaluation of the jejunum and ileum was carried out using an Axioskop-40 optical microscope (Leica, Wetzlar, Germany). Images were collected with an Axiocam 503 color camera (Carl Zeiss, Oberkochen, Germany) and the program used for morphometric measurements in image captures was Zen 3.2 (Carl Zeiss, Oberkochen, Germany).

The length of the villi and the depth of the Lieberkühn crypts were measured with the objective of ×10 magnification in all animals in the study: 10 measurements in the ileum and 10 measurements in the jejunum.

The ratio between villus height and crypt depth was calculated by dividing villus height by crypt depth (V/C ratio).

Cell counts of intraepithelial lymphocytes and intraepithelial goblet cells were performed in all study animals (10 fields in jejunum and 10 fields in ileum) with the objective ×63 magnification, each field consisting of a surface area of 3200 μm^2^.

All morphometric measurements and cell counts were blindly performed by the same researcher.

### 2.8. Immunohistochemistry

Multiple 5 µm thick sections of the paraffin samples were obtained for the study of IgA immunocytochemistry in jejunum and ileum tissue.

Samples were deparaffinized and rehydrated, and endogenous peroxidase activity was inhibited with a 1.5% H_2_O_2_ solution in methanol for 30 min. Samples were pretreated with 10% pronase in TBS (Sigma-Aldrich, St. Louis, MO, USA) for 12 min for antigen retrieval. Subsequently, samples were rinsed in TBS for (3 × 5 min) and incubated for 30 min with 100 μL of blocking solution per slide at 20 °C in a humid chamber. Subsequently, samples were incubated for 1 h at 37 °C with the primary antibody (goat anti-pig IgA, Byl, Montgomery, TX, USA) diluted 1:3000 in TBS. Secondary antibody (biotin-conjugated rabbit anti-goat, Dako, Carpinteria, CA, USA), diluted 1:200 in TBS, was incubated for 30 min at 20 °C. The avidin–biotin–peroxidase complex technique was used with the Vectastain^®^ Elite ABC kit (Vector, Newark, CA, USA), which was applied for 1 h at 20 °C. Positive development was detected using 3,3′-diaminobenzidine tetrahydrochloride (Dako, Carpinteria, CA, USA) for 5 min. Sections were stained with Mayer’s hematoxylin, dehydrated and mounted. The number of IgA-producing cells in the intestinal lamina propria was counted using a Zeiss Axioskop 40 microscope (Carl Zeiss, Oberkochen, Germany) with a Zeiss Axiocam 503 color camera (Carl Zeiss, Jena, Germany).

Immunolabeled cells were counted in 10 consecutive non-overlapping fields in jejunum and 10 fields in ileum with the objective of ×40 and were expressed as number of cells/36,000 μm^2^.

### 2.9. Statistical Analysis

All analyses were carried out using the statistical software package SPSS v. 25 (SPSS Inc., Chicago, IL, USA). The normality of the data was tested using a Kolmogorov–Smirnov test. Since the data were non-normal, a Kruskal–Wallis test was used for the non-parametric comparison of multiple samples, with a Mann–Whitney U test for two comparisons of independent samples. A discriminant function analysis (DFA) was used to perform a data reduction, considering only the functions for Wilks lambda *p* < 0.05. Moreover, correlations among parameters were calculated as partial correlations controlled for group and sampling. Having only one data point for each parameter for each group, two comparison strategies were used: a single sample Student *t*-test was performed to compare the individual data of each experimental group with the data obtained in the same buildings with animals of the same origin the previous year. To compare one experimental group with the other, it was considered that when there was a difference greater than one standard deviation of the annual data between the results of each group, it could be considered a significant difference, as previously published [28]. The frequency of each cause of mortality in each group was studied using a chi-square test with subsequent adjusted residuals analysis (AR). Differences between the observed and expected frequency were considered to exist when AR was >1.96 (higher frequency than expected) or <−1.96 (lower frequency than expected).

In all tests, a difference was considered significant when *p* < 0.05 and a trend difference when *p* > 0.05 and <0.1.

## 3. Results

### 3.1. Growth Performance, Mortality and Antibiotic Consumption

The weaning weight of the piglets was 5.70 kg in vaccinated piglets and 5.71 kg in unvaccinated piglets, and the final weight was 30.95 kg in the vaccinated and 28.89 kg in the unvaccinated piglets. The average daily gain (ADG) was 0.351 in the vaccinated group compared to 0.331 in the unvaccinated. As well as the feed conversion rate was 1.726 in the vaccinated compared to 1.823 in the non-vaccinated group (Table 3).

When looking at the differences between groups, we found more than one standard deviation of difference between groups for the final weights, ADG and FCR, so we can assume that the differences are significant. In fact, while there was a 1.4 s.d. difference in the final weight and a difference 1.7 s.d. in the ADG, for the FCR, we observed a 2.7 s.d. difference; so, we can consider the difference to be very significant. When comparing the data of each group with the annual data of the same buildings by means of one sample Student *t*-test, significant differences were found in the FCR of the V group (*p* = 0.03) but not for the CON group (*p* = 0.162) and especially in the final weight of the V group (V *p* < 0.001 vs. CON *p* = 0.192, respectively). The final weight of the V group was 2.55 kg higher than the average of all groups reared in that nursery in the twelve months prior to the test (average of 28.40 ± 1.47).

The results for mortality are shown in Table 4, segregating the causes of death with the highest frequency over the total (diarrhea, encephalitis, Glässer’s disease and hernias).

Mortality was higher in vaccinated piglets (4.47%) compared to unvaccinated piglets (3.34%). However, there were differences between observed and expected frequencies for diarrheal deaths with higher-than-expected frequency in the CON group (V = 21.6% vs. CON = 35% of total deaths, AR = 2.1, *p* < 0.001). A difference between the expected and observed frequency was also found for animals that died or were slaughtered due to inguinal, scrotal or umbilical hernias, with a higher frequency in the V group (V = 21.6% vs. CON = 0% of total deaths, AR = 4.5, *p* < 0.001). Mortality due to Glasser’s disease and Streptococcus-related encephalitis was numerically different but did not differ between groups (V = 21.6% vs. CON = 14.5% of total deaths, AR = 1.3, NS for Glässer’s disease and V = 7.2% vs. CON = 12%, AR = 1.1, NS for encephalitis).

When comparing the difference between the cause-specific mortalities found in each group, we again observe more than one s.d. difference between groups for diarrhea and hernias but not for Glässer’s disease.

Regarding antibiotic consumption on the farm, the group of vaccinated piglets had lower levels, with 58.4 mg/PCU of gentamicin, 169 mg/PCU of tiamulin and 251.6 mg/PCU of doxycycline consumed; while unvaccinated piglets consumed of 84.4 mg/PCU of gentamicin, 228.7 mg/PCU of tiamulin and 281.8 mg/PCU of doxycycline (Table 5).

### 3.2. Intestinal Integrity

**Figure 1:**
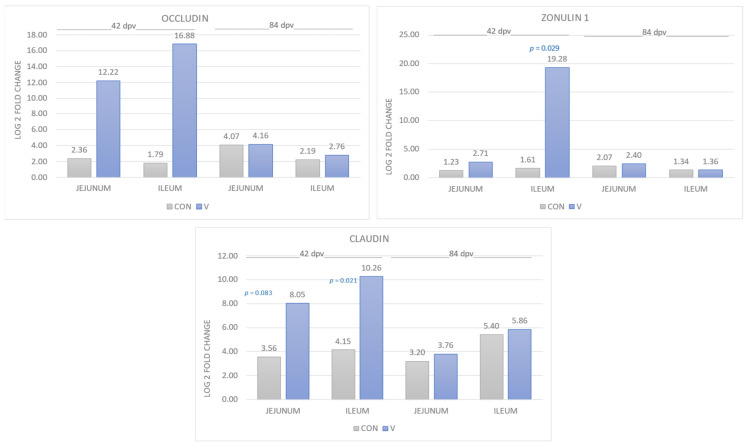
Quantifications of the three TJ proteins, OCCL, ZON and CLA assayed in the jejunum and ileum, at both 42 and 84 dpv. The bars represent the mean ± standard error of the mean (SEM) and the data labels are the mean. The significance level *p* appears as superscript in each biomarker. CON = control group and V = vaccinated group.

The gene expressions of the three TJ proteins are shown in Figure 1. The OCL was increased in the V group compared to the CON group, both in jejunum and ileum, at 42 dpv, with a 1000-fold difference in jejunum and approximately a 32,000-fold difference in ileum. However, at 84 dpv, the vaccinated animals also showed higher values, but the differences between both groups were not significant. Regarding the ZON, greater gene expression was found in the V group than in the CON group, both in jejunum and ileum at different ages. Furthermore, in the ileum at 42 dpv, significant differences were found (*p* = 0.029) with a higher expression in the V group, and a 208,000-fold difference in increased gene expression. Finally, CLA presented higher gene expression in the V group compared to the CON group in both jejunum and ileum at 42 dpv and 84 dpv. Furthermore, in the youngest animals (42 dpv), the differences between groups were statistically significant in the ileum (64-fold higher; *p* = 0.021) and a statistical trend was observed in the jejunum (22-fold higher; *p* = 0.083).

### 3.3. Immune Stimulation

**Figure 2:**
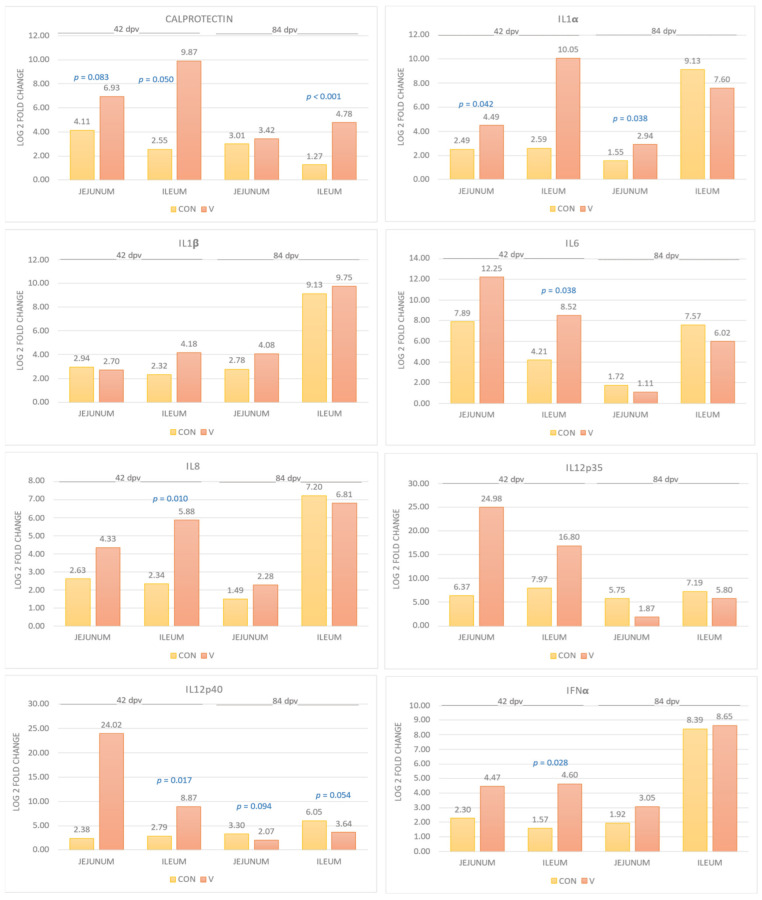

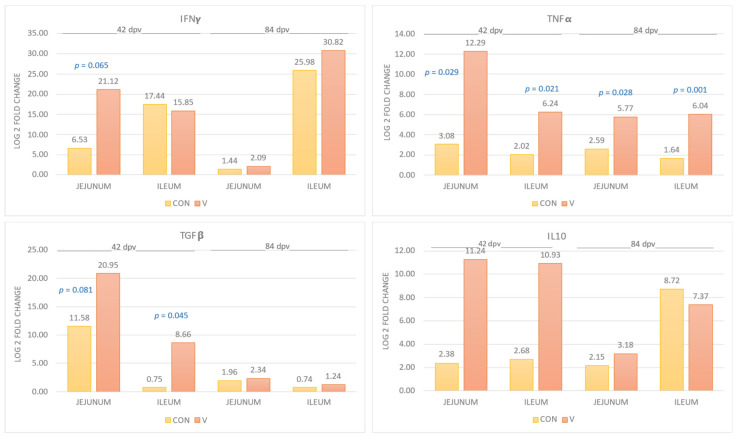
Quantifications of the gene expression of interleukins: calprotectin, IL1α, IL1β, IL6, IL8, IL12p35, IL12p40, IFNα, IFNγ, TNFα, TGFβ and IL10 assayed in the jejunum and ileum, at both 42 and 84 dpv. The bars represent the mean ± standard error of the mean and the data labels are the mean. The significance level *p* appears superscript in each biomarker. CON = control group and V = vaccinated group.

The quantifications of the gene expression of cytokines according to age and type of sample are shown in Figure 2.

CAL showed a statistical trend (*p* = 0.083) in favor of the V group compared to the CON group in the jejunum and at 42 dpv, with a 7-fold increase. While in the ileum, greater expression was observed with statistically significant differences both at 42 dpv (160-fold; *p* = 0.050) and at 84 dpv (11-fold; *p* < 0.001) in the V group (Figure 2).

In relation to IL1α, there was a statistically significant greater expression in the V group compared to the CON group, both at 42 days dpv (*p* = 0.042) and at 84 dpv (*p* = 0.038) in the jejunum, with a 4- and 2.5-fold difference in expression, respectively. On the contrary, IL1β did not show significant differences in any group (42–84 dpv) either in the jejunum or in the ileum.

Regarding IL6 (*p* = 0.038) and IL8 (*p* = 0.010), statistically significant results were observed only in the ileum at 42 dpv with higher gene expression in the V group compared to CON. The difference between groups was 20-fold for IL6 and 42-fold for IL8.

In the cases of IL12p35 and IL12p40, different patterns were present depending on the age of the animals. At 84 dpv, higher gene expression values were observed in the CON group compared to the V group, both in the jejunum and in the ileum (2.5- and 5.2-fold, respectively), with IL12p40 showing a statistical trend (*p* = 0.094) in the jejunum and statistical significance in the ileum (*p* = 0.054). On the contrary, at 42 dpv, both IL12p35 and IL12p40 showed higher gene expression in the V group compared to the CON group, and the latter with statistically significant results in favor of the V group (64-fold higher; *p* = 0.017) in ileum.

In relation to IFNα, greater gene expression was observed in the V group compared to the CON group in both jejunum and ileum at both ages. Furthermore, at 42 dpv in ileum, significant differences, i.e., 8-fold higher, were observed between groups (*p* = 0.028).

Regarding IFNγ, a statistical trend was observed only in the jejunum at 42 dpv with higher gene expression in the V group compared to the CON group (*p* = 0.065). The difference in gene expression was 24,800-fold higher in the V group.

Regarding TNFα, there was greater gene expression in the V group compared to the CON group in all cases, with statistically significant differences: at 42 dpv in jejunum (592-fold; *p* = 0.029) and in ileum (19-fold; *p* = 0.021), and at 84 dpv in jejunum (8-fold; *p* = 0.028) and in ileum (21-fold; *p* = 0.001).

In the case of TGFβ, higher gene expression was observed in the V group compared to the CON group in both jejunum and ileum (at 42 and 84 dpv). However, the jejunum at 42 dpv showed a statistical trend (*p* = 0.081) in favor of the V group, and in the ileum at 42 dpv, statistically significant results were observed (256-fold higher; *p* = 0.045).

Finally, IL10 had higher gene expression in the V group compared to the CON group in the jejunum at both ages, but in the ileum only at 42 dpv, without significant results.

### 3.4. Histomorphology

The morphometry results are shown in Table 6.

The highest villi were observed in the V group both in the jejunum (42 dpv and 84 dpv) and in the ileum at 42 dpv. However, in the ileum at 84 dpv, a statistical trend was detected (*p* = 0.091), namely that these longer villi were of the CON group.

**Figure 3:**
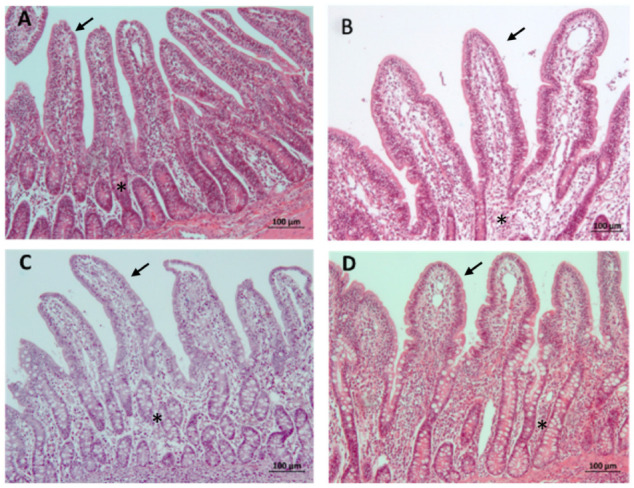
Villi measured in the jejunum at 42 dpv (**A**) and 84 dpv (**B**) as well as in the ileum at 42 dpv (**C**) and 84 dpv (**D**) in vaccinated animals. In the jejunum, longer villi (arrow) are observed in vaccinated animals in (**A**,**B**), and crypts (asterisk) with lesser depth were identified in (**A**,**B**). In the ileum of vaccinated animals, the villi (arrow) are longer, and the depth of the crypts (asterisk) is less in both (**C**,**D**). Hematoxylin–eosin stain. All images have the same magnification; the bar size in all images is 100 μm.

Overall, the V group showed lower crypt depths than the CON group. In the jejunum, this effect was only observed at 84 dpv (*p* = 0.058), while in the ileum it was detected at both 42 dpv and 84 dpv (*p* = 0.002). An example of villus images appears in Figure 3.

Regarding the V/C ratio, significant differences were only found in the jejunum at 84 dpv with a lower ratio in the CON group (*p* = 0.027).

### 3.5. Intraepithelial Lymphocytes, Goblet Cells and IgA-Producing Cells

**Figure 4:**
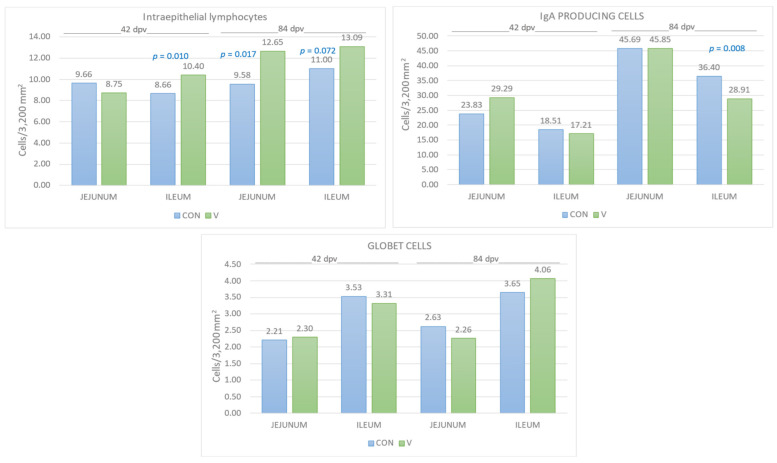
Results of cells count of intraepithelial lymphocytes, goblet cells, and IgA-producing cells in the jejunum and ileum, at both 42 and 84 dpv. The bars represent the mean ± standard error of the mean and the data labels are the mean. The significance level *p* appears as superscript in the bars. CON = control group and V = vaccinated group.

The results of cells count of intraepithelial lymphocytes, goblet cells and IgA-producing cells appear in Figure 4.

Regarding intraepithelial lymphocytes, they appear in greater quantities in the V group, showing significant differences in the jejunum at 84 dpv (*p* = 0.017) and in the ileum at 42 dpv (*p* = 0.010). A statistical trend is also observed in the ileum at 84 dpv (*p* = 0.072) in favor of the V group.

**Figure 5:**
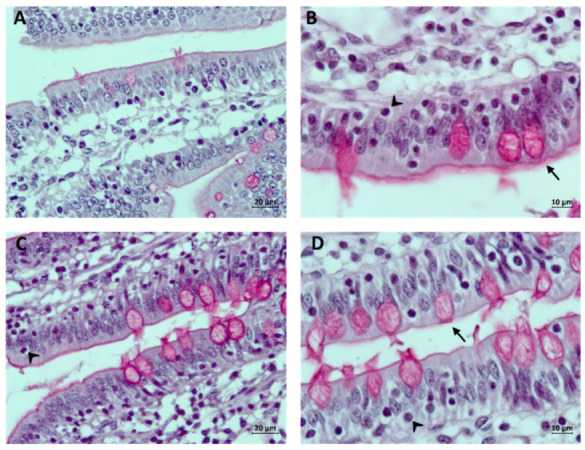
Microscopic images for counting intraepithelial lymphocytes and goblet cells in the jejunum at 42 dpv (**A**) and 84 dpv (**B**) as well as in the ileum at 42 dpv (**C**) and 84 dpv (**D**) in vaccinated animals. The number of goblet cells was similar in vaccinated and non-vaccinated animals. However, more goblet cells (arrow) were observed in (**B**,**D**) than in (**A**,**C**). Intraepithelial lymphocytes (arrowhead) were observed in greater numbers in vaccinated animals in (**B**–**D**). Periodic acid–Schiff stain. The bar size is 20 μm in images (**A**,**C**); in images (**B**,**D**), the bar size is 10 μm.

The goblet cell count showed similar values in both jejunum and ileum at both ages in both the CON and V groups. An example of microscopic images where intraepithelial lymphocytes and goblet cells were observed appears in Figure 5.

**Figure 6:**
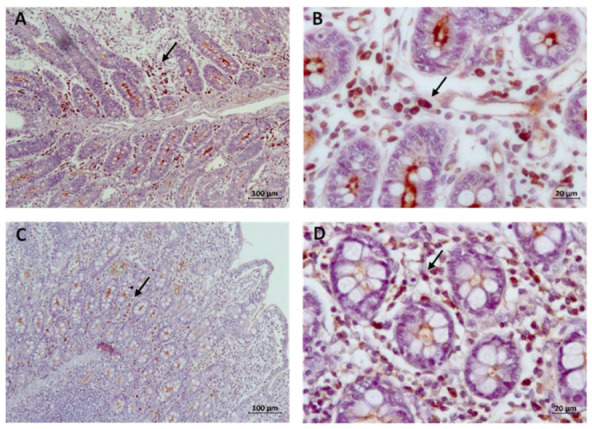
Microscopic images for counting IgA-producing cells in the jejunum at 42 dpv (**A**) and 82 dpv (**B**) as well as in the ileum at 42 dpv (**C**) and 84 dpv (**D**). There are more IgA-producing cells (arrow) in (**B**,**D**) than in (**A**,**C**). Mayer’s hematoxylin stain. The bar size is 100 μm in images (**A**,**C**); in images (**B**,**D**), the bar size is 20 μm.

Finally, the count of IgA-producing cells showed greater cellularity in the V group in the jejunum at 42 dpv and lower cellularity in the ileum at 84 dpv (*p* = 0.008). An example of microscopic images where IgA-producing cells were observed appears in Figure 6.

### 3.6. Discriminant Function Analysis

The DFA of all parameters analyzed (histomorphology, gene expression in jejunum and ileum and IgA-producing cell density) yielded a non-significant Wilks’ lambda (*p* = 0.564) with only one function, although the group membership assignment capacity was 93.3%.

When using histomorphology data alone, Wilks’ lambda showed a significance of *p* = 0.019 and a classification ability to group membership of 96.3%. However, when taking TJ protein and cytokine gene expression together, Wilks’ lambda was not significant (*p* = 0.636), but when discriminating by tissue, it was not significant for ileum (*p* = 0.286) but significant for jejunum (*p* = 0.024). In any case, its ability to classify into a group was greater than in 94% in all cases. And finally, DFA using the IgA-producing cell density data yielded a significance for Wilks’ lambda of *p* = 0.011, with a group assignability of 65.6%, but with 75% of the V group being well assigned.

When the data were segmented according to time post-vaccination, significant Wilks’ lambda were observed at 42 days for the analysis of histomorphology (*p* = 0.045) with a classification capacity of 100%. And at 84 days, all parameters (*p* = 0.031) had a sorting ability of 87.5%, gene expression in jejunum (*p* = 0.002) had a sorting ability of 100% and IgA-producing cell density (*p* = 0.004) had an assignment to group capability of 93.8%.

These results suggest that the effect of vaccination is more significant on histomorphology and the number of IgA-producing cells than on immune stimulation in ileum or jejunum and that the effects on tissue occur earlier than those of other markers.

## 4. Discussion

The elimination of tools to control enteric diseases in pigs, such as antibiotics or zinc oxide, has led the swine industry to look for other strategies such as improved management and the use of vaccines. It is necessary to understand how these vaccines work in order to improve their use and vaccination protocols. And above all, it is necessary to understand the factors that are involved in post-weaning diarrhea. One of the key elements could be post-weaning stress, associated with a prolonged and transient response in the gene expression of pro- and anti-inflammatory cytokines and IgA-producing cells in the intestine [4].

In the present work, we studied the gene expression of different biomarkers, the production of immune cells and the histological morphology at the intestinal level to assess the effect of vaccination against ETEC in piglets in the medium and long term (42 and 84 days post vaccination, respectively). So far, several studies have been published on the effects in the short term, generally considered up to 21 days post vaccination [7,9,23] or in the very long term, understood as up to the end of the animal’s life [24], but not in the medium term, which is the focus of our study.

At a productive level, we observe that vaccination against ETEC is beneficial in the animals studied, since those in the V group have a better ADG (0.351 kg/day vs. 0.331) and FCR (1.726 vs. 1.823) compared to those in the CON group. Various studies show productive data in animals vaccinated against *E. coli* with a higher ADG compared to unvaccinated animals, both at 14 days [27] and at 21 days post vaccination [7,23], and even at the end of finihing [29].

Furthermore, it has been shown that vaccination against *E. coli* reduces the incidence of diarrhea and the rate of mortality [30], as well as a reduction in the use of antibiotics [5]. In our study, we had a higher mortality in the V group (4.47%) than in the CON group (3.34%); however, considering the causes of mortality, in the V group, there were fewer deaths due to diarrhea (0.97%) than in the CON group (1.17%). As has already been observed in previous studies [5], the consumption of antibiotics was lower in the V group than in the CON group, especially regarding those antibiotics utilized for enteric processes, such as gentamicin (58.4 mg/PCU in the V group versus 84.4 mg/PCU in the CON group).

Regarding the quantification of the three TJ proteins (OCL, ZON and CLA), we were able to observe a greater gene expression in the V group compared to the CON group in the jejunum and ileum, demonstrating better intestinal integrity in the piglets that were vaccinated against *E. coli*, with this effect being much more evident at 42 dpv. Several studies demonstrate the increase in the expression of genes encoding tight junction proteins in dietary supplements [31], observing a better intestinal barrier effect due to the increase in the proliferation and differentiation of intestinal epithelial cells [9,32], protecting against the colonization of pathogenic bacteria and paracellular macromolecular transmission [13]. Furthermore, it has been shown that in piglets orally vaccinated against *E. coli* and subjected to diets with zinc oxide, intestinal integrity is better conserved with a greater gene expression of OCL, ZON and CLA soon after vaccination (6, 8 and 15 dpv) [27]. The absence of differences in the 84-day sampling suggests that unvaccinated animals regain homeostasis in mRNA production for TJ proteins and this reduces the between-group differences in the gene expression of these proteins.

There are very useful biomarkers as indicators of inflammation in various pathologies and cellular infiltration, such as CAL [33]. The quantification of calprotectin, both as a protein and as mRNA, increases when there is local immunological stimulation, such as a possible response to the invasion of the vaccine strains of *E. coli* in the intestine [9]. This response to immune stimulation has not only been detected at an early age (15 dpv) [27] but we were also able to observe it in the medium and long term in piglets vaccinated against *E. coli* (42 and 84 dpv) in greater amounts than in the CON group in the ileum, possibly due to the recruitment of antigen-presenting cells, such as neutrophils and macrophages, which essential for producing an immune response against the *E. coli* vaccine. This fact suggests that the immune effect of the vaccine can last for a long time at the tissue level. In fact, in the ileum and jejunum of *E. coli*-vaccinated animals tested at the end of fattening, an increased presence of inflammatory infiltrate was observed as a consequence of cell recruitment. In this situation, animals with the highest inflammatory infiltrate had the best productive results, indicating that this infiltrate was not pathological but the result of vaccination [24]. The presence of antigen-presenting cells in this infiltrate would undoubtedly explain the increased amount of CAL in vaccinated animals.

Pro-inflammatory cytokines may participate in increasing intestinal permeability, while anti-inflammatory cytokines control the duration and severity of inflammation. In several studies, an increase in the levels of proinflammatory cytokines has been observed in pigs challenged with *E. coli* ETEC [34], suggesting that the intestinal mucosal immune system is regulated by the presence of ETEC [19]. In our study, we observed a first cytokine immune response to vaccination in a differentiated manner in favor of the V group. When observing this effect in the medium term (42 dpv) in the jejunum, it is evident that IL1α, IFNγ, TNFα and TGFβ present a greater response immune. Similarly, in the medium term in the ileum, we observed that the cytokines with the highest expressions in the V group were IL6, IL8, IL12p40, IFNα, TNFα and TGFβ. This is consistent with the results observed at the end of the anima’s life, where increased gene expression against IFNγ, TGFβ and TNFα can be seen to be increased in vaccinated animals [24]. This suggests that the effect of vaccination is prolonged throughout the productive life of the animals, from almost the time of vaccination until slaughter at the slaughterhouse. Interestingly, the long-term studies were carried out with a parenteral vaccine that in the short term (21 days) does not produce an evident increase in cytokines [9], inducing a state of inactivation until a second contact with *E. coli.* The coincidence of the increased cytokine expression profile in the vaccinated animals with our medium- and long-term results would indicate that once the response is established, whether through an oral or parenteral vaccine, it is long lasting and keeps the animals protected.

Previous studies indicate that an increase in the levels of IFNγ and TGFβ may indicate a state of immune activation at the time of sampling after exposure to the F4 antigen of *E. coli* [9,27], and in our case, this vaccine effect continues to be observed in the medium term (42 dpv).

In this study, we also observed that in the ileum there is greater immune stimulation with a greater production of IL6, which promotes the differentiation of B cells and IL8, which stimulates the migration of neutrophils to the site of inflammation to fight against possible pathogens [19]. Previous studies demonstrated that the presence of ETEC could increase the production of IL1α, TNFα, IL6, IL8 and even IL12p35 [22].

On the other hand, in the long term (84 dpv), it is more difficult to find an immune response to vaccination against *E. coli*. However, in the jejunum, we observed that IL1α, IL12p40 and TNFα show a statistically significant greater response in the V group, and the same occurs in the ileum with IL12p40 and TNFα.

Of the interleukins, TNFα shows greater gene expression in all cases in our study, both in jejunum and ileum at 42 and 84 dpv, with significant differences in favor of the V group. This cytokine is the one that reacts best to the stimulus of the F4 antigen of *E. coli* and maintains its long-term immunity effect [35]. The local expressions of IL1, IL6 and TNFα were reported mainly after the exposure of pigs to a bacterial or viral infection and even in humans in the form of inflammatory bowel diseases [4].

Regarding histomorphology, in our study, we observed a greater height of intestinal villi and a lower depth of the crypts in the V group, therefore resulting in a better V/C ratio. This observation is directly related to the integrity of the mucosa in vaccinated piglets, which is of great importance for both nutrient absorption and intestinal health [13]. This finding was demonstrated in previous research [9] using different food additives [13,36]. These histomorphometry results are consistent with those obtained from the gene expression of cytokines used as biomarkers resulting from the effect of local stimulation on the intestinal mucosa.

Altogether, a greater density of intraepithelial lymphocytes was observed in the ileum and jejunum both in the medium and long term (42 and 84 dpv, respectively) after *E. coli* vaccination in piglets. This finding allows us to assess that there is a better response by the GALT (intestine-associated lymphatic tissue) after antigenic stimulation such as vaccination. Intraepithelial lymphocytes are directly related to a great capacity for immune surveillance [37], simultaneously participating in innate and adaptive immune regulation and in the maintenance of intestinal homeostasis [19]. This finding is closely related to the increase in CAL as a biomarker in the V group, also observed in this research, since both parameters increase with local stimulation.

Regarding the quantification of IgA-producing cells, greater cellularity was observed in the V group in the jejunum in the medium term after vaccination (42 dpv). This fact is related to what was observed in the jejunum in the short term after oral vaccination (8, 10, 15 and 24 dpv) in previous studies [7,23,27], and at 21 dpv in the ileum [9]. The presence of IgA-producing cells in the GALT at the time of vaccination or natural infection, as well as the presence of secretory IgA, is crucial to prevent the adhesion of *E. coli* to the intestinal mucosa [10]. Furthermore, the oral route is the natural stimulation route for the production of IgA as a local protective element against gastroenteric pathogens [35], although there are variations in what has been observed in other investigations depending on the intestinal tract studied, possibly due to the difference in density from GALT [9].

However, the long-term effects studied (84 dpv) on the production of IgA-producing cells resulted in an increase in cells compared to 42 dpv, but no differences were observed between groups in the jejunum, while in the ileum, the CON group showed greater cellularity compared to the V group. This result could be due to a loss of local humoral immunity in the long term, and a second oral stimulation with a vaccine was possibly necessary to continue the production of immunoglobulins in the intestine.

It is well known that the anti-inflammatory cytokines TGFβ and IL10 improve epithelial permeability and block the negative effects of *E. coli* infection [12]. Furthermore, both are essential to stimulate IgA production at the intestinal level and maintain mucosal tolerance between immunity and intestinal homeostasis [18]. We can observe that there were differences between study groups; in terms of TGFβ at 42 dpv, there were greater gene expressions with more significant differences in the V group in both jejunum and ileum. On the other hand, the gene expression of this cytokine at 84 dpv was very low and hardly any differences were seen between study groups. Something similar occurred with IL10, where at 42 dpv, there was greater gene expression, and at 84 dpv, the production decreased; it was higher in the CON group in the ileum. This could be another reason why at 84 dpv there were either no differences between groups in regard to IgA-producing cells or there was even greater production levels in the CON group.

When we compare the DFA results with previously published results at 21 days post vaccination with the same vaccine, it was observed that at 3 weeks post vaccination, DFA histomorphology, TJ protein and cytokine gene expression and IgA-producing cell density show a very significant Wilks’ lambda [9]. In this study, taking all data together, we did not find any significance. And while in the 21-day study, gene expression was found to be significant across all tissues, at 42 and 84 days, only in jejunum was there a significant differentiation with respect to the biomarkers. Interestingly, as observed in this work, there was a clear effect on histomorphology from 3 weeks post vaccination [9] to 12 weeks post vaccination, or even until the end of the animal’s life, as already documented for parenteral vaccination [24]. However, for the biomarkers of integrity and immunity, there was an immediate effect at 21 days; this effect was no longer evident at 42 days, but there was a further stimulation of the immune system at 84 days. This suggests that after the initial effect of vaccination there is a stabilization phase, probably followed by a booster effect due to contact with the pathogen throughout the nursery period. This would also be consistent with the very high production of IL10 and TGFβ at day 42 in the vaccinated group which subsequently decreased.

This research provides new information on the immune response that is generated in the body of piglets challenged with *E. coli* through the natural route of infection by observing the medium- and long-term effects after vaccination in different intestinal tracts. The response of different cytokines was studied in detail through biomarkers and cellular markers and the histomorphometry of the intestinal mucosa in order to understand the patterns of immune responses in piglets after *E. coli* vaccination. However, more research will be needed to fully understand the process.

## 5. Conclusions

Oral vaccination against *E. coli* in fattening piglets produces an evident immune response, which is manifested even in the medium and long term after the challenge. Growth performance (AGD and TI) in vaccinated animals was better than in non-vaccinated ones. TJ proteins (OCL, ZON and CLA) were shown in greater quantities in vaccinated animals, being more evident in the ileum at 42 dpv. Most of the interleukins studied, specifically CAL, IL1α, IL6, IL8, IL12p40, IFNα, IFNγ, TNFα and TGFβ, showed greater gene expression in vaccinated animals, especially at 42 dpv in the jejunum and/or ileum. Vaccinated piglets showed greater length of villi in the jejunum and ileum and greater intraepithelial lymphocyte density both in the medium and long term, especially at 84 dpv. The V group showed greater intestinal integrity and immune stimulation compared to the CON group.

## Figures and Tables

**Table 1:** Composition of the feed supplied during the test.

Pre-Starter		Starter	
Raw Material	Content (%)	Raw Material	Content (%)
Maize	29.82	Maize	32.71
Flaked maize, wheat and barley	19.90	Barley	13.36
Wheat	14.53	Wheat	24.53
Oat groats	5.85	Rye oats	5.95
Soybean meal	6.17	Soybean meal	13.17
Soy protein	6.00	Soy protein	1.94
Fish meal	1.90	Fish meal	0.78
Acid whey	3.27	Carbonate	0.66
Whey re-fatted whey	2.50	Porcine protein hydrolysate	1.22
Whey bran	2.50	Vitamin–mineral corrector *	0.29
Porcine protein hydrolysate	2.50	Carbonate	0.51
Vitamin–mineral corrector *	0.95	Monocalcium phosphate	0.92
Monocalcium phosphate	0.92	Amino acids	1.98
Amino acids	1.88	Mycotoxin sequestrant	0.19
Mycotoxin binder	0.15	Rehydrating agent	0.04
Water	0.97	Water	1.30
Salt	0.09	Salt	0.36

* The vitamin–mineral corrector supplied the following per kg complete diet. Vitamins: vitamin A, 13,000 I.U.; vitamin D3 I.U. 2000; vitamin E, 40 mg; vitamin K3, 2 mg; vitamin B1, 1.2 mg; vitamin B2, 5 mg; vitamin B6/pyridoxine hydrochloride, 3 mg; vitamin B12, 0.036 mg; niacinamide, 25 mg; D-calcium pantothenate, 20 mg; biotin, 0.11 mg; betaine anhydrous, 110 mg. Trace elements or trace element compounds: manganese (manganese oxide) 40 mg; iron (iron (II) sulphate monohydrate), 125 mg; copper (copper (II) sulphate pentahydrate), 130 mg; iodine (potassium iodide), 0.7 mg; selenium (sodium selenite), 0.3 mg. Digestives: 6-phytase EC 3.1.3.26 F.T.U. 500. Antioxidants: Butylated hydroxytoluene (BHT), 0.482 mg; propyl gallate (E 310), 0.004 mg. Carrier: fine wheat bran, barley. The piglets were fed pre-starter feed for 3 weeks after weaning and a starter feed from the 4th week onwards until the end of the nursery period.

**Table 2:** Primers for occludin (OCL), zonulin 1 (ZON), claudin 1 (CLA), calprotectin (CAL), IL-1β, IL-6, IL-8, IL-10, IL-12p35, IL-12p40, TNF-α, IFN-α, IFN-γ, TGF-β and β-Actin (housekeeper gene) used in this work.

Gene	Primer Forward (5′ to 3′)	Primer Reverse (5′ to 3′)
*OCL*	5′-TTGCTGTGAAAACTCGAAGC-3′	5′-CCACTCTCTCCGCATAGTCC-3′
*ZON*	5′-CACAGATGCCACAGATGACAG-3′	5′-AGTGATAGCGAACCATGTGC-3′
*CLA*	5′-ACCCCAGTCAATGCCAGATA-3′	5′-GGCGAAGGTTTTGGATAGG-3′
*CAL*	5′-AATTACCACGCCATCTACGC-3′	5′-TGATGTCCAGCTCTTTGAACC-3′
*IFN-α*	5′-CCCCTGTGCCTGGGAGAT-3′	5′-AGGTTTCTGGAGGAAGAGAAGGA-3′
*IFN-γ*	5-TGGTAGCTCTGGGAAACTGAATG-3′	5′-GGCTTTGCGCTGGATCTG-3′
*TNF-α*	5′-ACTCGGAACCTCATGGACAG-3′	5′-AGGGGTGAGTCAGTGTGACC-3′
*IL-12p35*	5′-AGTTCCAGGCCATGAATGCA-3′	5′-TGGCACAGTCTCACTGTTGA-3′
*IL-12p40*	5′-TTTCAGACCCGACGAACTCT-3′	5′-CATTGGGGTACCAGTCCAAC-3′
*IL-10*	5′-TGAGAACAGCTGCATCCACTTC-3	5′-TCTGGTCCTTCGTTTGAAAGAAA-3′
*TGF-β*	5′-CACGTGGAGCTATACCAGAA-3′	5′-TCCGGTGACATCAAAGGACA-3′
*IL-8*	5′-GCTCTCTGTGAGGCTGCAGTTC-3′	5′-AAGGTGTGGAATGCGTATTTATGC-3′
*IL-1α*	5′-GTGCTCAAAACGAAGACGAACC-3′	5′-CATATTGCCATGCTTTTCCCAGAA-3′
*IL-1β*	5′-AACGTGCAGTCTATGGAGT-3′	5′-GAACACCACTTCTCTCTTCA-3′
*IL-6*	5′-CTGGCAGAAAACAACCTGAACC-3′	5′-TGATTCTCATCAAGCAGGTCTCC-3′
*β-actin*	5′-CTACGTCGCCCTGGACTTC-3′	5′-GATGCCGCAGGATTCCAT-3′

**Table 3:** Results of growth performance for each group. V group = vaccinated group; CON group = control group.

	V Group	CON Group	ΔCON-V	s.d.	*p*-Value
Weaning weight	5.70 kg	5.71 kg	0.01	0.101	NS
Final weight	30.95 kg	28.89 kg	2.06	1.470	<0.05
ADG	0.351 g/d	0.331 g/d	0.020	0.012	<0.05
FCR	1.726 kg/kg	1.823 kg/kg	0.097	0.036	<0.05

ΔCON-V = difference between the data of groups V and CON in absolute numbers; s.d. = standard deviation of the data of all groups reared in the nursery 12 months before the test.

**Table 4:** Results of total mortality by group and specific mortality due to diarrhea and meningitis/encephalitis. V group = vaccinated group and CON group = control group.

	V Group	CON Group	ΔCON-V	s.d.	*p*-Value
Mortality	4.47%	3.34%	1.13	0.59	<0.05
Diarrhea mortality	0.97%	1.17%	0.2	0.151	<0.05
Glässer’s disease	0.97%	0.48%	0.49	0.501	NS
Encephalitis mortality	0.32%	0.40%	0.08	0.09	NS
Hernia	0.97%	0%	0.97	0.12	<0.005

ΔCON-V = difference between the data of groups V and CON in absolute numbers; s.d. = standard deviation of the data of all groups reared in the nursery 12 months before the test.

**Table 5:** Results of antibiotic consumption: gentamicin, tiamulin and doxycycline. V group = vaccinated group and CON group = control group.

	V Group	CON Group
Gentamicin	58.4 mg/PCU	84.4 mg/PCU
Tiamulin	169 mg/PCU	228.7 mg/PCU
Doxycycline	251.6 mg/PCU	281.8 mg/PCU

**Table 6:** Histomorphology results of jejunum and ileum: villus height, crypt depth and ratio V/C, shown in the jejunum and ileum, at both 42 and 84 dpv. CON = control group and V = vaccinated group.

			CON	V	*p*-Value
JEJUNUM	42 dpv	Villus height (μm)	394.51	422.93	NS
		Crypt Depth (μm)	256.73	291.11	NS
		V/C Ratio	1.56	1.48	NS
	84 dpv	Villus height (μm)	452.93	500.80	NS
		Crypt Depth (μm)	365.93	304.28	0.058
		V/C Ratio	1.25	1.67	0.027
ILEUM	42 dpv	Villus height (μm)	300.20	321.39	NS
		Crypt Depth (μm)	245.23	224.55	NS
		V/C Ratio	1.27	1.48	NS
	84 dpv	Villus height (μm)	435.68	391.33	0.091
		Crypt Depth (μm)	335.26	275.31	0.002
		V/C Ratio	1.31	1.43	NS

## Data Availability

The data that support the findings of this study are available on request from the corresponding author.
